# School children growth monitoring program in the state of Qatar: Observations from two survey rounds in 2016–17 and 2019–20

**DOI:** 10.1002/puh2.52

**Published:** 2023-01-12

**Authors:** Mohammed H. Al‐Thani, Salah A. Alyafei, Kholoud A. Al‐Mutawa, Shamseldin A. H. Khalifa, Amit Mishra, Benjamin V. Poovelil, Azza A. Abdellatif, Amine A. Toumi, Suresh B. Kokku

**Affiliations:** ^1^ Public Health Department Ministry of Public Health Doha Qatar

**Keywords:** children, growth monitoring, malnutrition, obesity, overweight, the state of Qatar

## Abstract

**Introduction:**

Growth monitoring surveys provide critical anthropometric data to monitor physical growth and various forms of malnutrition among school age children. In the beginning, growth monitoring programs were introduced to identify the extent of undernutrition among children, which were later considered equally useful in the identification of overweight and obesity among school age children. Observing the shifts in weight categories among school age children provides an important insight to design targeted interventions for improving growth and development of children.

**Methodology:**

The study used growth monitoring survey data among 5–19‐year school children of two academic years (2016–17 and 2019–20) in Qatar where 2016–17 survey included 186,986 students, whereas 2019–20 survey included 215,279 students. A total of 7514 unique records of students aged 5–14 years available in both survey rounds were included in the final analysis. This study documented shift in BMI‐*z*‐scores to ascertain the movement of students among obese, overweight, normal, thinness, and severe thinness categories. Python version 3.9.5 was used for data analysis along with a pairwise comparison between each of BMI‐*z*‐score shift to evaluate the effects of specific shifts in BMI‐*z*‐score category.

**Results:**

Overall, the proportion of overweight and obese category of students increased from 44% in 2016–17 to 49.3% in 2019–20 with a decrease in the proportion of students in normal BMI‐*z*‐score category (from 48.8% to 47.8%) and severe thinness and thinness category (from 7.3% to 3%) between two rounds of growth monitoring survey. Statistically significant shifts in BMI‐*z*‐score categories were noted for students of different age groups, gender, and nationality.

**Conclusions:**

Shift from normal BMI‐*z*‐score to obese and overweight category is a cause of concern and an opportunity to develop appropriate interventions. The significant shift among different categories needs to be investigated further to identify associated reasons to effectively develop interventions.

## INTRODUCTION

Growth monitoring is one of the important components of community‐based child health services, which includes routine anthropometric measurements to detect abnormal growth patterns followed by appropriate interventions to ensure optimal growth and development [1]. Growth monitoring is advocated globally as one of the key child survival and primary health‐care strategies. Weight gain is an important growth indicator under the age of five and growth monitoring acts as an excellent tool for assessing the growth and development of children [2, 3]. The most important issue in growth monitoring is not the position of the child on the growth curve but the direction of their growth to diagnose their health and nutritional status [4]. It is well known that malnutrition and consequent complications could be prevented through regular monitoring of the growth of children and the implementation of growth promotion activities referred to as growth monitoring and promotion (GMP).

The concept of GMP was introduced in the mid‐1980s with an emphasis to design health promotion programs based on the growth monitoring survey results. The proposed activities included growth monitoring survey, early disease detection, treatment, nutrition counseling, and supplementation to improve individual child nutritional outcomes and reduce child deaths [5, 6, 7]. It was believed that the GMPs are beneficial as it detects the problem and acts as a vehicle for health promotion [8]. Recognizing the importance of growth monitoring for child survival, GMPs were introduced in many countries and as of today about 178 countries use GMP for essential services related to children [9].

Growth monitoring surveys provide critical anthropometric data to monitor physical growth among school age children annually and help in identification of trends in various forms of malnutrition [10]. This periodical dataset is also an indirect measure of child feeding practices and helps to inform policies around nutrition and child development [11].

GMPs have highlighted child nutrition status as key development indicator and have had great impact on reducing severe malnutrition in many countries [12]. Although GMPs were introduced with an intent to identify the extent of undernutrition among children, these were found equally crucial in understanding the magnitude of overweight and obesity among the school age children. Childhood nutrition status has its impact on health in adults, as the childhood obesity increases chances of obesity in adulthood by five times [13].

The World Health Organization (WHO) estimated that the prevalence of childhood overweight and obesity is increasing each year in all regions of the world and there are about 39 million overweight and obese children under the age of 5 years and over 340 million between 5 and 19 years of age. The prevalence of overweight and obesity among children and adolescents aged 5–19 years has increased from 4% in 1975 to over 18% in 2016 affecting boys and girls equally [14].

Overweight and obesity have complex multifactorial etiopathogenesis, and a comprehensive strategy for its prevention, surveillance, and management is required to effectively address these conditions [15, 16]. Some countries are now increasingly facing double burden of nutrition‐related health issues where ongoing burden of undernutrition and micronutrient deficiencies exist alongside the epidemic of overweight and obesity [17].

Recognition of childhood obesity as a major public health problem has led to the development of an international growth standard for surveillance and monitoring of school‐aged children and adolescents by WHO in 2007 [18]. The WHO expert group suggested to use BMI‐for‐age charts up to 19 years for an assessment of thinness, overweight, obesity, and stunting in school‐aged children and adolescents [19].

Tracking of overweight and obesity has gained significant traction in past two decades globally due to, in parts, its linkages with higher mortality rates as compared to underweight and success of childhood obesity prevention programs in some settings [20, 21]. Many countries, such as the United States, Canada, India, Australia, and New Zealand, have put in place systems for the surveillance of childhood obesity [22, 23, 24, 25, 26]. WHO European Childhood Obesity Surveillance Initiative compiles childhood obesity surveillance data for European countries to inform policies and practices in the region [27]. Among Gulf Cooperation Council (GCC), some studies have measured childhood obesity in Bahrain, Kuwait, Saudi Arabia, Qatar, and UAE [28, 29, 30, 31]. However, comprehensive national representative survey data for childhood obesity is available only from Kuwait [32].

Realizing the rising burden of obesity among children, the state of Qatar started GMP for school going children aged between 5 and 19 years during academic year 2014–15 in public schools, which was later extended to private schools as well. The GMP was initiated as part of the state of Qatar's national nutrition and physical activity action plan to promote healthy lifestyle among school children. The program aimed to detect overweight, obese, and underweight status among adolescents and aspired to build a database and surveillance system on weight and height distribution in schools using new WHO growth monitoring charts.

This current study is an analysis of a subset of growth monitoring data collected in 2016–17 and 2019–20 from schools in the state of Qatar. The objective of this study is to document changes in BMI‐*z*‐scores among various categories of school children in two survey rounds to identify shifts in specific student population groups and implement appropriate interventions in the state of Qatar.

## MATERIALS AND METHODS

### Study design, setting and population

This is a retrospective analysis of BMI‐*z*‐scores among a cohort of 7514 school children aged 5–14 years. This cohort was identified from the growth monitoring survey conducted in 2016–17 and 2019–20 in the state of Qatar. The methodology for the collection of growth monitoring data from schools in the state of Qatar has been described in earlier publications [33].

The Ministry of Education and Higher Education (MEHE) governs the education system in the state of Qatar, and schools are divided into four levels, that is, preprimary (below 5 years), primary (5–9 years), preparatory (10–14 years), and secondary schools (15–19 years). During academic year 2016–17, a total of 247,920 students were enrolled in 570 schools, whereas in 2019–20, 276,975 students were studying in 673 schools. Growth monitoring survey is conducted in all schools since 2014–15 except in preprimary schools where the growth of child is monitored through facility‐based child growth monitoring program and in those schools where school nurses are not available.

In 2016, the state of Qatar had a total population of 2.4 million with approximately 308,700 individuals in the age group 5–19 years. This study was conducted using cross‐sectional survey data from growth monitoring program of two academic years 2016–17 and 2019–20. The 2016–17 survey included 186,986 students aged 5–19 years from 332 school campuses, whereas the 2019–20 survey included a total of 215,279 students from 350 schools.

### Data collection methods and tools

All school nurses were trained on collecting anthropometric measurements using WHO methods. A standard data collection paper form was used in both survey years, containing information about name of student, identification number, date of birth, contact details of parents, grade, nationality, and height and weight measurements along with the date of measurement. These paper records were later computerized by nurses, and an annual report on this data was published by Ministry of Public Health (MOPH).

Both surveys were conducted in respective school premises during schooling hours. Height of the survey participant was recorded in centimeters to the nearest decimal place using SECA model 206 CM roll‐up measuring tape, and weight was recorded in kilogram to the nearest decimal place using the SECA model 813 digital floor scale. Both measurements were taken without shoes.

Baseline data for this study was drawn from the records of the students who participated in the growth monitoring survey during the academic year 2019–20. These student records were then matched with the student records available in the 2016–17 survey using a common unique identifier. Records of the students that were not available in either of the survey rounds or where records had inconsistencies in age, gender, nationality, and grade levels were excluded. In addition, records of 15–19‐year students were also excluded due to very small number of participants. A total of 7514 unique student records from two survey rounds were included in the final analysis.

### Data analysis

Deidentified data in Microsoft Excel (2021) format for both survey years was obtained from the public health department, MOPH to compute the BMI‐*z*‐scores. The BMI was calculated by dividing weight in kilograms by height in meters squared (kg/m^2^). Depending upon the age and sex of the student, BMI (kg/m^2^) was converted into the BMI‐*z*‐score in accordance with guidelines formulated by WHO [34]. Using BMI‐*z*‐score, five different categories were formed, including severe thinness (BMI‐*z*‐score <−3), thinness (BMI‐*z*‐score ≥−3 to <−2), normal (BMI‐*z*‐score ≥−2 to <1), overweight (BMI‐*z*‐score ≥+1 to <+2), and obese (BMI‐*z*‐score ≥+2) [35]. For analyzing data, Python version 3.9.5 with pandas, numpy, and statsmodels libraries was used [36]. A pairwise comparison between each of BMI‐*z*‐score shift category was conducted to evaluate the effects of specific shifts in BMI‐*z*‐score category. This effect was evaluated using a McNemar test of homogeneity with exact statistics. The McNemar test is a nonparametric test for paired dichotomous data. In simple words, this technique is used to find a change in proportion for the paired data. *p*‐Value of <0.05 was statistically significant.

### Ethical consideration

Growth monitoring survey is an annual public health activity conducted jointly by MOPH and MEHE in the state of Qatar to monitor the growth of children in schools. All ethical procedures were followed during data collection process as per the MOPH, MEHE, and primary health‐care corporation policies (reference documents: TS/W/686, TS/AGA/597). The deidentified data obtained for this study is owned by MOPH.

## RESULTS

### Participant characteristics

The study included 7514 students in the final analysis, of which 51.4% were male students, and 48.6% were female students. About 50.6% of the students were Qatar nationals, and remaining 49.4% belonged to other nationalities. Around 38.8% of the students belonged to the 5–9‐year age group, whereas 61.2% were in the 10–14‐year age group (Table 1).

**TABLE 1:** Basic characteristics of school children surveyed for growth monitoring data in the schools in Qatar in 2019–20

Population characteristics	*n*	%
Total students	7514	100
Sex		
Male	3863	51.4
Female	3651	48.6
Nationality		
Qatar	3799	50.6
Non‐Qatar	3715	49.4
Age group (years)		
5–9	2913	38.8
10–14	4601	61.2

### BMI‐*z*‐score categories

**FIGURE 1:**
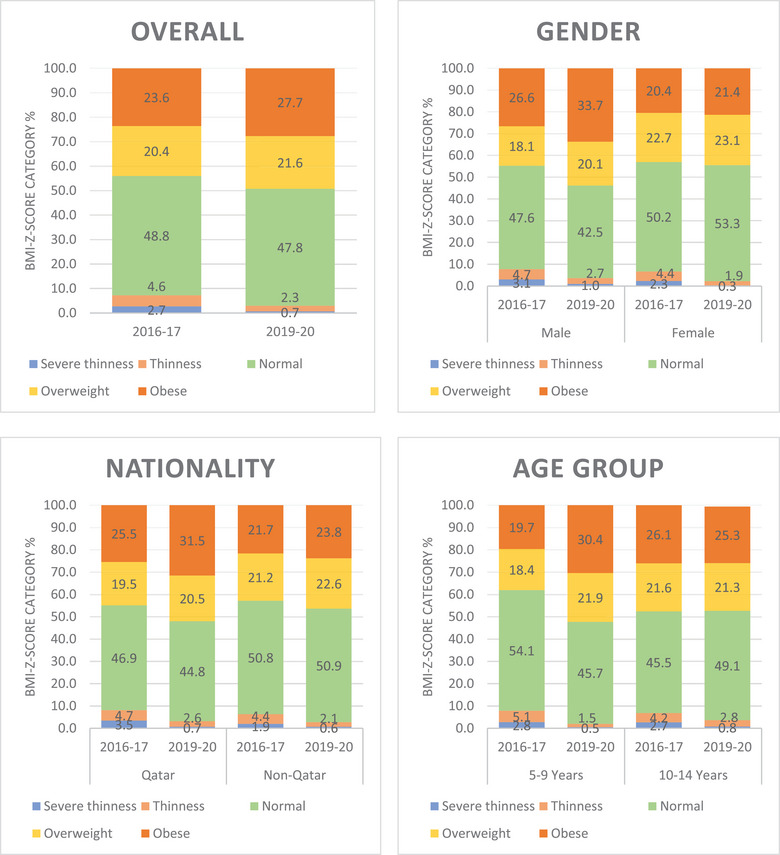
BMI‐*z*‐score comparisons across age group, gender, and nationality among school children surveyed in Qatar in 2016–17 and 2019–20

Overall, the proportion of overweight and obese category of students increased from 44% (obese 23.6% and overweight 20.4%) in 2016–17 to 49.3% (obese 27.7% and overweight 21.6%) in 2019–20 (Figure 1). During the same period, the proportion of students in normal BMI‐*z*‐score decreased from 48.8% in 2016–17 to 47.8% in 2019–20. There was also a decrease noted in the proportion of severe thinness and thinness category students from 7.3% (thin 4.6% and severe thin 2.7%) to 3% (thin 2.3% and severe thin 0.7%) between two rounds of growth monitoring survey. Among individual categories, there was 4.1% increase in obese category (highest increase, *p* < 0.05) followed by 1.2% increase in overweight category and 2.3% decrease in thinness category (highest decrease, *p* < 0.05) followed by 2% decrease (*p* < 0.05) in severe thinness category.

Between 2016–17 and 2019–20, statistically significant (*p* < 0.05) shift was noted among male students across all BMI‐*z*‐score categories, where the proportion of obese male students increased from 26.6% to 33.7%, and overweight from 18.1% to 20.1%, whereas normal BMI‐*z*‐score students decreased from 47.6% to 42.5%, thin students from 4.7% to 2.7%, and severe thin students from 3.1% to 1%. Among female students, statistically significant (*p* < 0.05) decrease was noted among a proportion of thin students from 4.4% to 1.9% and severe thin students from 2.3% to 0.3%. The proportion of obese students increased (*p* < 0.05) both in Qatar national (from 25.5% to 31.5%) and non‐Qatar students (from 21.7% to 23.8%). In addition, both Qatar national and non‐Qatar students have shown statistically significant reduction in proportions of thinness and severe thinness. Among 5–9‐year age group students, statistically significant (*p* < 0.05) shift was observed in all categories, where proportions of obese students increased from 19.7% to 30.4%, overweight from 18.4% to 21.9%, whereas the proportion of normal BMI‐*z*‐score students reduced from 54.1% to 45.7%, thin students reduced from 5.1% to 1.5%, and severe thin students reduced from 2.8% to 0.5%. Among 10–14‐year age group students, statistically significant (*p* < 0.05) increase in proportion of normal BMI‐*z*‐score students was noted from 45.5% to 49.1%, and a decrease in proportions of thin students from 4.2% to 2.8% (*p* < 0.05) and severe thin students from 2.7% to 0.8% (*p* < 0.05) was observed. Though the proportions of overweight and obese students reduced in this age group as well, this shift was statistically not significant (Table S1).

**FIGURE 2:**
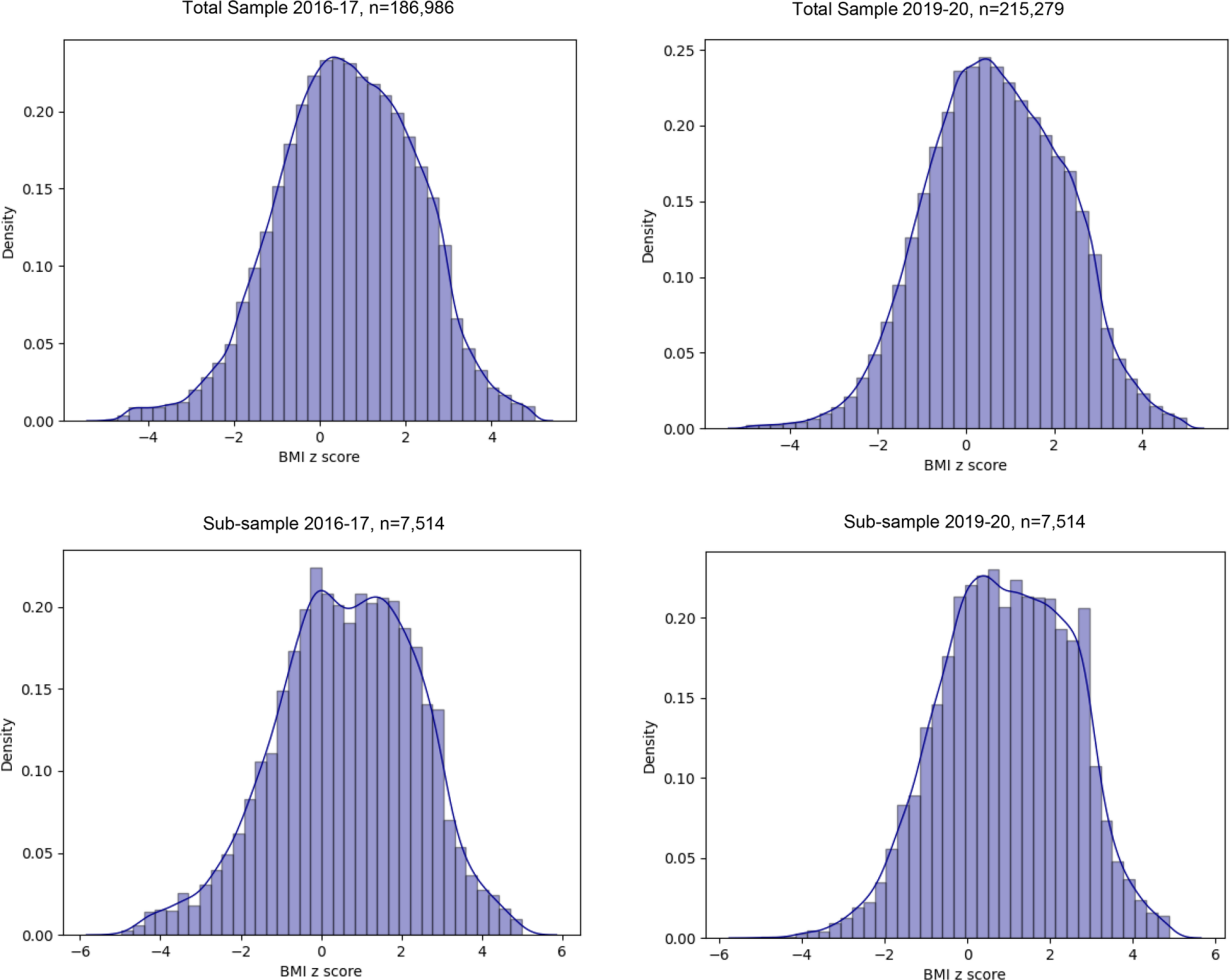
Distribution of BMI‐*z*‐scores in 2016–17 and 2019–20 among total and subsample of school children

The trends suggest rise in overweight and obesity among school age children in the state of Qatar, and students from all age groups have moved in different categories during the study period. There is an overall decrease in the proportion of thinness and severe thinness category of students across all age groups (Figure 2).

**FIGURE 3:**
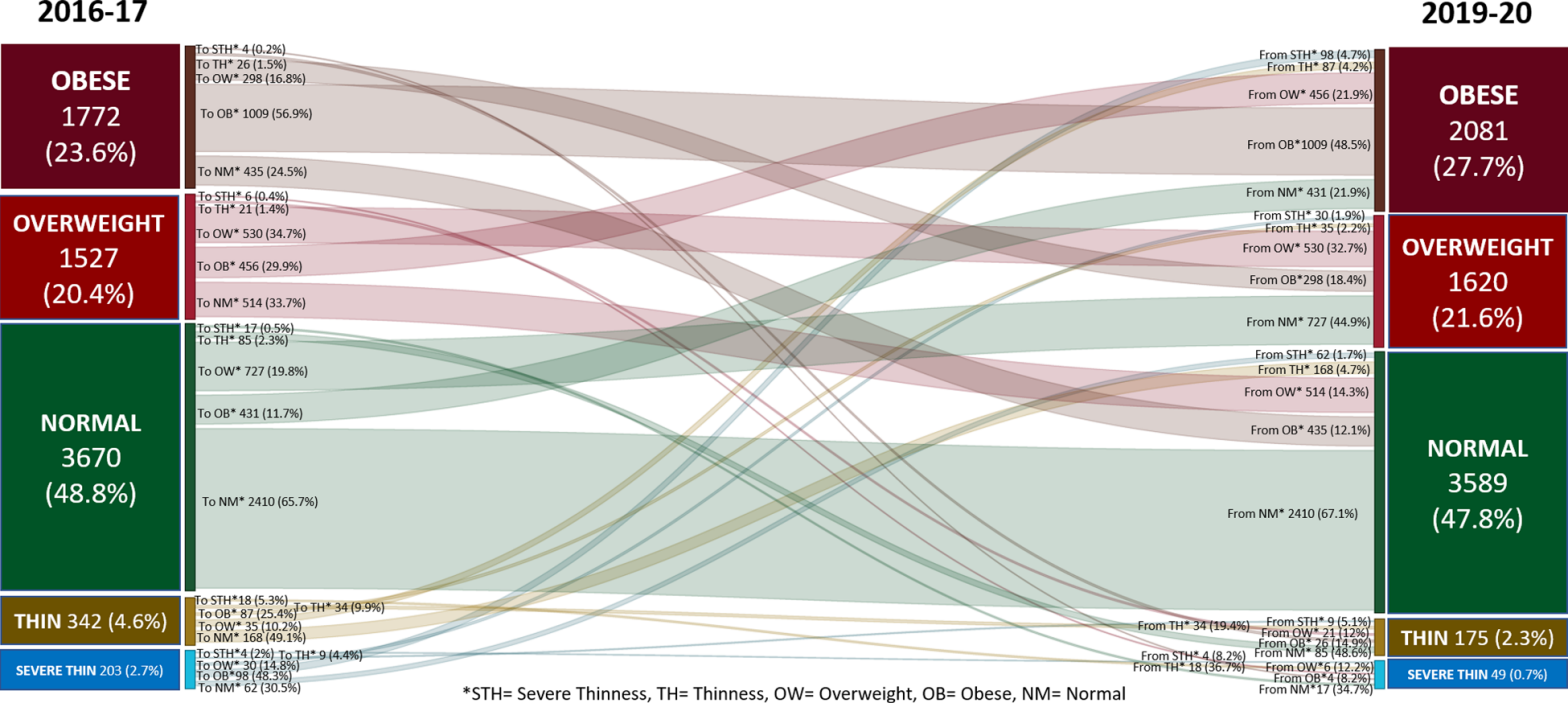
Shifting of BMI‐*z*‐score categories (2016–17 to 2019–20), *n* = 7514

The results from the overall shift in categories suggest that in comparison with 2016–17, in 2019–20, more students shifted to obese category from overweight (6%, *p* < 0.001) and normal BMI‐*z*‐score (5.7%, *p* < 0.001), as compared to obese students shifting to overweight (3.9%, *p* < 0.001), and normal BMI‐*z*‐score categories (5.7%, *p* < 0.001) (refer to Tables S2 and S3). This trend in shift was observed across 5–9‐year age group students, Qatar national and non‐Qatar students and male students with exception of female students and 10–14‐year age group students where a reverse trend was noted (Figure 3).

During study period, a total of 32% students remained in normal BMI‐*z*‐score, whereas 13.4% stayed as obese, and another 7% remained as overweight without changing their BMI‐*z*‐scores. Female students with normal BMI‐*z*‐score had high retention percentages (35.6%) as compared to male students (28.6%). In addition, 34.7% of non‐Qatar students maintained their normal BMI‐*z*‐score as compared to Qatar national students (29.4%). Normal BMI‐*z*‐score retention was higher among 5–9‐year‐old students (34.3%) compared to 10–14‐year age group students (30.6%).

## DISCUSSION

This study is an attempt to examine the growth monitoring data of 2016–17 and 2019–20 from the state of Qatar to understand the BMI‐*z*‐score category shifts among the school age children. Earlier studies have reported high BMI rates among the school children in the state of Qatar [33]. However, there is limited information available about the shifts in weight categories within various groups of school children during different time periods.

Our results show that obesity is increasing in both Qatar national and non‐Qatar students in general, particularly among the male students and students in 5–9‐year age group. However, female students and students from 10‐ to 14‐year age group have shown decline in obesity. Though the current study does not explore the reasons of such change, these shifts are important to understand to further explore the associated causes for such shifts among different groups of students.

The significant shifts from normal category to overweight and obese categories are concerning and could be attributed to multiple reasons such as unhealthy diets, physical inactivity, and relatively low breastfeeding rates in the state of Qatar [37, 38, 39]. Childhood obesity increases the risk of noncommunicable diseases in later life [40], and shift toward increased BMI category is also associated with worsening of cardiometabolic risk factors [41].

The state of Qatar has a growing and large subpopulation (66%) of younger people (<30‐year old) and according to estimates, about 57% of obese (BMI ≥ 30 kg/m^2^) and overweight (BMI 25–30 kg/m^2^) people are less than 30‐year old [42]. Unhealthy eating habits are common among adolescents in the state of Qatar, and about three fourth of them consume sugar‐sweetened drinks and skip breakfast more than three times a week [43]. Physical inactivity is an important risk factor for obesity among children, and more than half of students in the state of Qatar spend more than 2 h per day watching television, surfing internet, or playing electronic games [44]. It was also reported that about 25% of students in the state of Qatar are not involved in any kind of physical activity, whereas 48% participated in physical activity for less than 1 h per day [45]. Data from Qatar national school survey reported that only about 35% of students performed 60 min of physical activity ≥3 days/week and according to Qatar Active Healthy Kids (QAHK) 2018 report only about 25% of children attain at least 60 min of moderate‐to‐vigorous physical activity daily [46].

Common causes of obesity, such as unhealthy diet and physical inactivity, are rooted in a set of complex social, psychological, technological, environmental, and economic factors. Given that the dietary and physical activity habits are shaped during the early years of life, it is important to inculcate the healthy behaviors at this age. Usually, children spend more than a decade of their life in the school, and this is an important entry point for imparting knowledge and skills around healthy lifestyle behaviors that have long‐term impact [47].

Qatar Public Health Strategy (2017–22) intends to educate people about making healthy lifestyle choices and provide them with greater options that encourage and assist changes in behaviors [48, 49]. The Qatar Nutrition and Physical Activity Action Plan (2017–22) is in‐line with the global and regional targets for improving maternal, infant, and young child nutrition in the country [50]. The plan has specific interventions to improve the school health such as regulation of unhealthy foods in schools, incorporation of nutrition into school curriculum, and improving infrastructure for physical activity programs. The action plan suggests interventions to place restrictions on marketing, advertising, and sponsorship for all fat/sugar‐rich foods and drinks to children across all media platforms. In addition, it recommends to progressively eliminate any subsidies by government on sugar and fat and increase taxes on sweetened soft drinks and beverages.

Realizing the promising impact of obesity reduction interventions among children, the Qatar National Health Strategy (2018–22) aspired to halt the rate of overweight and obesity amongst children and adolescents aged 5–19‐year old. Though the current public health policies have facilitated in halting the rates of obesity among children to certain extent, the reversal of trends needs active engagement of various stakeholders such as parents, schools, media, government agencies, and community representatives [51, 52].

Examination of eating practices of children and the availability and utilization of physical activity opportunities at homes and in schools needs further review [53]. Collaboration between relevant stakeholders will facilitate in devising the strategies that reduce the children's inactivity time and promote physical activity through various approaches [54]. Family support, including improving parental awareness and knowledge of healthy diet and physical activity, can significantly affect the health outcomes among children. In our study, various students moved from overweight and obese category to normal BMI‐*z*‐score categories, whereas other students have moved from normal BMI‐*z*‐score to obese and overweight category. The determinants of these shifts need to be studied to develop appropriate interventions.

This study has certain strengths such as large multiethnic sample across the country and growth measurement data collected by trained nurses using WHO‐approved standards. The limitations of the study include lack of information on other parameters to identify determinants for shift in various categories. We acknowledge that there might be a chance of selection bias inherent to retrospective cohort studies.

## CONCLUSION

The state of Qatar has a high prevalence of childhood obesity and halting the rate of overweight and obesity amongst children and adolescents has been one of the major targets of Qatar National Health Strategy (2018–22). This study has identified some of the important shifts in BMI‐*z*‐scores among various groups of students that could be helpful in designing targeted interventions. The study calls for further research to identify underlying determinants of shifts in the categories to develop appropriate intervention for preventing childhood obesity.

## AUTHOR CONTRIBUTIONS

*Conceptualization and methodology*: Mohammed H. Al‐Thani, Salah A. Alyafei, Kholoud A. Al‐Mutawa, Amit Mishra. *Data analysis and validation*: Shamseldin A. H. Khalifa, Benjamin V. Poovelil, Amine A. Toumi. *Writing—original draft, review, and editing*: Amit Mishra, Azza A. Abdellatif, Suresh B. Kokku. *Supervision*: Mohammed H. Al‐Thani, Salah A. Alyafei, Kholoud A. Al‐Mutawa, Shamseldin A. H. Khalifa.

## CONFLICT OF INTEREST

The authors declare no conflict of interest.

## ETHICS STATEMENT

All ethical procedures were followed during data collection process as per the MOPH, MEHE, and PHCC policies (Reference documents: TS/W/686, TS/AGA/597).

## Supporting information

Tables S1–S3

## Data Availability

The data that supports the findings of this study is available on request from the corresponding author. The data is not publicly available due to privacy or ethical restrictions.
