# Supplementary material for: School children growth monitoring program in the state of Qatar: Observations from two survey rounds in 2016–17 and 2019–20
Source: Public Health Chall. 2023 Jan 12;2(1):e52. doi: 10.1002/puh2.52 (PMC12039658; doi:10.1002/puh2.52)
Supplement: Supplementary file 1 — Tables S1–S3 [file PUH2-2-e52-s001.docx]

| **Table-1: Proportion of BMIS-z-score categories across age group, gender and nationality [n(%)]** | | | | |
| --- | --- | --- | --- | --- |
| **Overall** | | | | |
| ***Survey Year*** | ***2016-17*** | | ***2019-20*** | |
| Total students | 7514 (100) | | 7514 (100) | |
| Obese | 1772 (23.58) | | 2081 (27.69)* | |
| Overweight | 1527 (20.37) | | 1620 (21.56) | |
| Normal | 3670 (48.80) | | 3589 (47.76) | |
| Thinness | 342 (4.55) | | 175 (2.33)* | |
| Severe thinness | 203 (2.70) | | 49 (0.66)* | |
| **Gender** | **Male** | | **Female** | |
| ***Survey Year*** | ***2016-17*** | ***2019-20*** | ***2016-17*** | ***2019-20*** |
| Total students | 3863 (100) | 3863 (100) | 3651 (100) | 3651 (100) |
| Obese | 1027 (26.59) | 1301 (33.68)* | 745 (20.41) | 780 (21.36) |
| Overweight | 700 (18.12) | 777 (20.11)* | 827 (22.65) | 843 (23.09) |
| Normal | 1837 (47.55) | 1642 (42.51)* | 1833 (50.21) | 1947 (53.33)* |
| Thinness | 180 (4.66) | 105 (2.72)* | 162 (4.43) | 70 (1.92)* |
| Severe thinness | 119 (3.08) | 38 (0.98)* | 84 (2.30) | 11 (0.30)* |
| **Nationality** | **Qatar national** | | **Non-Qatar** | |
| ***Survey Year*** | ***2016-17*** | ***2019-20*** | ***2016-17*** | ***2019-20*** |
| Total | 3799 (100) | 3799 (100) | 3715 (100) | 3715 (100) |
| Obese | 967 (25.45) | 1198 (31.53)* | 805 (21.67) | 883 (23.77)* |
| Overweight | 740 (19.48) | 779 (20.51) | 787 (21.18) | 841 (22.64) |
| Normal | 1782 (46.91) | 1700 (44.75) | 1888 (50.82) | 1889 (50.85) |
| Thinness | 179 (4.71) | 97 (2.55)* | 163 (4.39) | 78 (2.10)* |
| Severe thinness | 131 (3.45) | 25 (0.66)* | 72 (1.94) | 24 (0.64)* |
| **Age Group** | **5-9 Years** | | **10-14 years** | |
| ***Survey Year*** | ***2016-17*** | ***2019-20*** | ***2016-17*** | ***2019-20*** |
| Total | 2913 (100) | 2913 (100) | 4601 (100) | 4601 (100) |
| Obese | 573 (19.67) | 886 (30.42)* | 1199 (26.06) | 1195 (25.34) |
| Overweight | 535 (18.37) | 638 (21.90)* | 992 (21.56) | 982 (21.34) |
| Normal | 1575 (54.07) | 1331 (45.69)* | 2095 (45.53) | 2258 (49.08)* |
| Thinness | 149 (5.11) | 45 (1.54)* | 193 (4.19) | 130 (2.83)* |
| Severe thinness | 81 (2.78) | 13 (0.45)* | 122 (2.65) | 36 (0.78)* |
| *p<0.05 compared to 2016-17 batch for every BMI category | | | | |

| **Table 2 Shift in BMI z-score for the students in Qatar across two survey rounds (2016-17 and 2019-20) [n (%)]** | | | | | | | | |
| --- | --- | --- | --- | --- | --- | --- | --- | --- |
|  | | | | **2019-20 batch** | | | | |
|  |  |  |  | **Obese** | **Overweight** | **Normal** | **Thinness** | **Severe Thinness** |
| **2016-17 Batch** | **Overall** | | Obese | 1009 (13.4) | 298 (3.97) ⱡ | 435 (5.79) ⱡ | 26 (0.35) | 4 (0.05) |
|  |  |  | Overweight | 456 (6.07) ⱡ | 530 (7.05) | 514 (6.84) | 21 (0.28) | 6 (0.08) |
|  |  |  | Normal | 431 (5.74) ⱡ | 727 (9.68) | 2410 (32.07) | 85 (1.13) | 17 (0.23) |
|  |  |  | Thinness | 87 (1.16) | 35 (0.47) | 168 (2.24) | 34 (0.45) | 18 (0.24) |
|  |  |  | Severe Thinness | 98 (1.3) | 30 (0.4) | 62 (0.83) | 9 (0.12) | 4 (0.05) |
|  | **Age groups** | **5-9** | Obese | 400 (13.73) | 68 (2.33) ⱡ | 104 (3.57) ⱡ | 1(0.03) | 0 (0.0) |
|  |  |  | Overweight | 231 (7.93) ⱡ | 191 (6.56) | 112 (3.84) | 0 (0.0) | 1 (0.03) |
|  |  |  | Normal | 193 (6.63) ⱡ | 352 (12.08) | 1000 (34.33) | 25 (0.86) | 5 (0.17) |
|  |  |  | Thinness | 31 (1.06) | 14 (0.48) | 86 (2.95) | 14 (0.48) | 4 (0.14) |
|  |  |  | Severe Thinness | 31 (1.06) | 13 (0.45) | 29 (1.0) | 5 (0.17) | 3 (0.10) |
|  |  | **10-14** | Obese | 609 (13.24) | 230 (5.0) | 331 (7.19) | 25 (0.54) | 4 (0.10) |
|  |  |  | Overweight | 225 (4.89) | 339 (7.37) | 402 (8.74) | 21 (0.46) | 5 (0.11) |
|  |  |  | Normal | 238 (5.17) | 375 (8.15) | 1410 (30.65) | 60 (1.30) | 12 (0.26) |
|  |  |  | Thinness | 56 (1.22) | 21 (0.46) | 82 (1.78) | 20 (0.43) | 14 (0.30) |
|  |  |  | Severe Thinness | 67 (1.46) | 17 (0.37) | 33 (0.72) | 4 (0.09) | 1 (0.02) |
|  | **Gender** | **Male** | Obese | 613 (15.87) | 154 (3.99) ⱡ | 238 (6.16) ⱡ | 18 (0.47) | 4 (0.10) |
|  |  |  |  |  |  |  |  |  |
|  |  |  | Overweight | 269 (6.96) ⱡ | 213 (5.51) | 202 (5.23) | 11 (0.28) | 5 (0.13) |
|  |  |  | Normal | 288 (7.46) | 377 (9.76) | 1108 (28.68) | 50 (1.29) | 14 (0.36) |
|  |  |  | Thinness | 61 (1.58 | 17 (0.44) | 66 (1.71) | 23 (0.60) | 13 (0.34) |
|  |  |  | Severe Thinness | 70 (1.81) | 16 (0.41) | 28 (0.72) | 3 (0.08) | 2 (0.05) |
|  |  | **Female** | Obese | 396 (10.85) | 144 (3.94)* | 197 (5.40)* | 8 (0.22) | 0 (0.00) |
|  |  |  | Overweight | 187 (5.12)* | 317 (8.68) | 312 (8.55) | 10 (0.27) | 1 (0.03) |
|  |  |  | Normal | 143 (3.92)* | 350 (9.59) | 1302 (35.66) | 35 (0.96) | 3 (0.08) |
|  |  |  | Thinness | 26 (0.71) | 18 (0.49) | 102 (2.79) | 11 (0.30) | 5 (0.14) |
|  |  |  | Severe Thinness | 28 (0.77) | 14 (0.38) | 34 (0.93) | 6 (0.16) | 2 (0.05) |
|  | **Nationality** | **Qatar** | Obese | 575 (15.14) | 141 (3.71) ⱡ | 232 (6.11) ⱡ | 17 (0.45) | 2 (0.05) |
|  |  |  | Overweight | 256 (6.74) ⱡ | 237 (6.24) | 231 (6.08) | 12 (0.32) | 4 (0.11) |
|  |  |  | Normal | 246 (6.48) ⱡ | 366 (9.63) | 1118 (29.43) | 43 (1.13) | 9 (0.24) |
|  |  |  | Thinness | 57 (1.50) | 16 (0.42) | 83 (2.18) | 17 (0.45) | 6 (0.16) |
|  |  |  | Severe Thinness | 64 (1.68) | 19 (0.50) | 36 (0.95) | 8 (0.21) | 4 (0.11) |
|  |  | **Non-Qatar** | Obese | 434 (11.68) | 157 (4.23)* | 203 (5.46)* | 9 (0.24) | 2 (0.05) |
|  |  |  | Overweight | 200 (5.38)* | 293 (7.89) | 283 (7.62) | 9 (0.24) | 2 (0.05) |
|  |  |  | Normal | 185 (4.98)* | 361 (9.72) | 1292 (34.78) | 42 (1.13) | 8 (0.22) |
|  |  |  | Thinness | 30 (0.81) | 19 (0.51) | 85 (2.29) | 17 (0.46) | 12 (0.22) |
|  |  |  |  |  |  |  |  |  |
|  |  |  | Severe Thinness | 34 (0.92) | 11 (0.30) | 26 (0.70) | 1 (0.03) | 0 (0.0) |

| **Table 3 Shift in BMI z-score Categories for students in various age groups according to their nationalities and gender [n(%)]** | | | | | | | | |
| --- | --- | --- | --- | --- | --- | --- | --- | --- |
| **2019-20** | | | | | | | | |
| **2016-17** |  |  |  | **Obese** | **Overweight** | **Normal** | **Thinness** | **Severe thinness** |
|  | **Male** | **5-9** | Obese | 253 (14.67) | 36 (2.09) ⱡ | 69 (4.0) ⱡ | 1 (0.06) | 0 (0.0) |
|  |  |  | Overweight | 147 (8.52) ⱡ | 79 (4.58) | 59 (3.42) | 0 (0.0) | 1 (0.06) |
|  |  |  | Normal | 133 (7.71) ⱡ | 207 (12.0) | 579 (33.57) | 17 (0.99) | 5 (0.29) |
|  |  |  | Thinness | 25 (1.45) | 9 (0.52) | 40 (2.32) | 10 (0.58) | 3 (0.17) |
|  |  |  | Severe Thinness | 27 (1.57) | 8 (0.46) | 15 (0.87) | 1 (0.06) | 1 (0.06) |
|  |  | **10-14** | Obese | 360 (16.84) | 118 (5.52) | 169 (7.90) | 17 (0.80) | 4 (0.19) |
|  |  |  | Overweight | 122 (5.71) | 134 (6.27) | 143 (6.69) | 11 (0.51) | 4 (0.19) |
|  |  |  | Normal | 155 (7.25) | 170 (7.95) | 529 (24.74) | 33 (1.54) | 9 (0.42) |
|  |  |  | Thinness | 36 (1.68) | 8 (0.37) | 26 (1.22) | 13 (0.61) | 10 (0.47) |
|  |  |  | Severe Thinness | 43 (2.01) | 8 (0.37) | 13 (0.61) | 2 (0.09) | 1 (0.05) |
|  | **Female** | **5-9** | Obese | 147 (12.37) | 32 (2.69) ⱡ | 35 (2.95)* | 0 (0.0) | 0 (0.0) |
|  |  |  | Overweight | 84 (7.07) ⱡ | 112 (9.43) | 53 (4.46) | 0 (0.0) | 0 (0.0) |
|  |  |  | Normal | 60 (5.05)* | 145 (12.21) | 421 (35.44) | 8 (0.67) | 0 (0.0) |
|  |  |  | Thinness | 6 (0.51) | 5 (0.42) | 46 (3.87) | 4 (0.34) | 1 (0.08) |
|  |  |  | Severe Thinness | 4 (0.34) | 5 (0.42) | 14 (1.18) | 4 (0.34) | 2 (0.17) |
|  |  | **10-14** | Obese | 249 (10.11) | 112 (4.55) | 162 (6.58) ⱡ | 8 (0.32) | 0 (0.0) |
|  |  |  | Overweight | 103 (4.18) | 205 (8.32) | 259 (10.52) | 10 (0.41) | 1 (0.04) |
|  |  |  | Normal | 83 (3.37) ⱡ | 205 (8.32) | 881 (35.77) | 27 (1.10) | 3 (0.12) |
|  |  |  | Thinness | 20 (0.81) | 13 (0.53) | 56 (2.27) | 7 (0.28) | 4 (0.16) |
|  |  |  | Severe Thinness | 24 (0.97) | 9 (0.37) | 20 (0.81) | 2 (0.08) | 0 (0.0) |
|  | **Qatar Students** | **5-9** | Obese | 229 (14.84) | 33 (2.14) ⱡ | 62 (4.02) ⱡ | 1 (0.06) | 0 (0.0) |
|  |  |  | Overweight | 134 (8.68) ⱡ | 83 (5.38) | 57 (3.69) | 0 (0.0) | 0 (0.0) |
|  |  |  | Normal | 112 (7.26) ⱡ | 192 (12.44) | 490 (31.76) | 17 (1.10) | 4 (0.26) |
|  |  |  | Thinness | 21 (1.36) | 7 (0.45) | 45 (2.92) | 4 (0.26) | 4 (0.26) |
|  |  |  | Severe Thinness | 20 (1.30) | 5 (0.32) | 16 (1.04) | 4 (0.26) | 3 (0.26) |
|  |  | **10-14** | Obese | 346 (15.34) | 108 (4.79) | 170 (7.54)* | 16 (0.71) | 2 (0.09) |
|  |  |  | Overweight | 122 (5.41) | 154 (6.83) | 174 (7.71) | 12 (0.53) | 4 (0.18) |
|  |  |  | Normal | 134 (5.94)* | 174 (7.71) | 628 (27.84) | 26 (1.15) | 5 (0.22) |
|  |  |  | Thinness | 36 (1.60) | 9 (0.40) | 38 (1.68) | 13 (0.58) | 2 (0.09) |
|  |  |  | Severe Thinness | 44 (1.95) | 14 (0.62) | 20 (0.89) | 4 (0.18) | 1 (0.04) |
|  | **Non-Qatar Students** | **5-9** | Obese | 171 (12.48) | 35 (2.55) ⱡ | 42 (3.07) ⱡ | 0 (0.0) | 0 (0.0) |
|  |  |  | Overweight | 97 (7.08) ⱡ | 108 (7.88) | 55 (4.01) | 0 (0.0) | 1 (0.0) |
|  |  |  | Normal | 81 (5.91) ⱡ | 160 (11.68) | 510 (37.23) | 8 (0.58) | 1 (0.07) |
|  |  |  | Thinness | 10 (0.73) | 7 (0.51) | 41 (2.99) | 10 (0.73) | 0 (0.0) |
|  |  |  | Severe Thinness | 11 (0.80) | 8 (0.58) | 13 (0.95) | 1 (0.07) | 0 (0.0) |
|  |  | **10-14** | Obese | 263 (11.22) | 122 (5.20) | 161 (6.87) ⱡ | 9 (0.38) | 2 (0.09) |
|  |  |  | Overweight | 103 (4.39) | 185 (7.89) | 228 (9.72) | 9 (0.38) | 1 (0.04) |
|  |  |  | Normal | 104 (4.43) ⱡ | 201 (8.57) | 782 (33.35) | 34 (1.45) | 7 (0.30) |
|  |  |  | Thinness | 20 (0.85) | 12 (0.51) | 44 (1.88) | 7 (0.30) | 12 (0.51) |
|  |  |  | Severe Thinness | 23 (0.98) | 3 (0.13) | 13 (0.55) | 0 (0.0) | 0 (0.0) |
